# Effect of dietary garlic (*Allium sativum*) on the zootechnical performance and health indicators of aquatic animals: A mini-review

**DOI:** 10.14202/vetworld.2023.965-976

**Published:** 2023-05-11

**Authors:** Daniel Leonardo Cala Delgado, Linda Lucia Castillo Caceres, Sebastián Alexander Cely Gómez, Aníbal Domínguez Odio

**Affiliations:** 1 Animal Science Research Group, Universidad Cooperativa de Colombia, Bucaramanga, Colombia; 2 Department of Veterinary Medicine, Universidad Cooperativa de Colombia, Bucaramanga, Colombia; 3 Dirección de Ciencia e Innovación, Grupo Empresarial LABIOFAM, La Habana, Cuba

**Keywords:** *Allium sativum*, aquaculture, crustacean, salmonid, tilapia

## Abstract

Considerable efforts have been made by modern aquaculture to mitigate the environmental damages caused by its practices while also attempting to improve the quality of the aquatic organisms by promoting alternatives, such as the use of natural products, like garlic (*Allium sativum*), and instead of chemical agents. Garlic has multiple properties, including antifungal, antibacterial, antiviral, antitoxic, and anticancer effects. In fish, the antiparasitic activity of garlic is one of the most reported effects in the literature, mainly using immersion baths for aquatic organisms. Using garlic also has an antimicrobial effect on the culture of aquatic organisms. Therefore, this review focuses on the impact of garlic on the health and production of aquatic organisms.

## Introduction

Aquatic animals and plant species have long been farmed for self-supply or commercial purposes. This practice is supported by over 2000 years of knowledge, thus determining its value as an income and protein source for people worldwide [1–4]. The consumption of aquatic products has been rapidly increasing [5], partially fueled by the world’s growing population and an increased interest in consuming healthy food [6]. However, this increase in production and consumption is not uniform globally. The geographic distribution of international trade shows that countries such as China, India, Egypt, Vietnam, Chile, and Norway account for 59% of the total fishing exports. In contrast, industrialized countries and geopolitical entities such as the United States, the European Union, and Japan import 71% of the international market’s fishing products [7, 8].

Urgent measures should be taken to increase, diversify, and optimize the current aquaculture production systems to meet the demand for protein consumption from aquatic species while lowering costs and minimizing resource utilization [2, 9].

The intensification of aquaculture practices leads to polyculture, high fish density per volume [10], and stress by overcrowding [1, 11], which favors the spread of infectious diseases [12]. Moreover, while each pathogen’s impact on aquatic health varies, they can all cause severe economic losses [13], because they affect the productive potential, require diagnosis and treatment, and can cause high mortality rates in aquatic species [14, 15].

These threats are managed by the use and abuse of synthetic chemical products, including antibiotics [16, 17]. These chemical products are harmful to the environment and human health because they bioaccumulate [18], accumulate in fish tissue [19], and promote the development of multidrug-resistant bacteria [20]. To reduce bacterial resistance, it is necessary to implement new strategies, which will avoid the use of traditional antimicrobial products [11] and favor the use of natural products [11, 21].

This environmental strategy encourages the generalized use of probiotics, prebiotics, postbiotics, and immunostimulants [22, 23], as well as the search for new natural alternatives regardless of their origin [24–28]. In addition, this strategy offers excellent opportunities to use herbal products, particularly those containing garlic (*Allium sativum*) [21, 29]. Therefore, this review focuses on the impact of garlic on the health and production of aquatic organisms.

## Natural Aquaculture Products

One of humanity’s oldest practices is the use of therapeutic plants [27, 30]. Despite the achievements in healthcare accomplished over time, active plant ingredients and their biological activities remain invaluable resources for humans [31]. Recently, ancestral and modern knowledge have merged. Ancestral knowledge helped identify the medicinal properties of leaves, roots, seeds, barks, fruits, and flowers [32], whereas modern knowledge incorporated new techniques to explain and optimize the application of leaves, roots, seeds, barks, fruits, and flowers [33].

The interest in natural products is rising in the veterinary sector; however, despite the similarities in the use of medicinal plants in animals and humans, manufacturing of natural products for animal use remains a significant shortcoming of this sector. In general, products for terrestrial production animals and those related to health and nutrition dominate the veterinary herbal product market [34]. A peculiar characteristic of this sector is that it is not a part of the domain of large international biopharmaceutical companies related to animal health based in the United States, Germany, and France [35].

Various plants have shown promising results in aquatic production [29], with more than 250 plant species identified belonging to 75 families and 32 orders [32, 36]. In aquaculture, plant-based products and byproducts are administrated or incorporated through intraperitoneal [37] or intramuscular injection [38], oral administration [39], and immersion [40].

Nevertheless, oral administration and incorporation through diet are the most recommended and agreed-on methods in aquatic species because they do not cause stress and allow simultaneous treatment of many organisms with minimal effort and cost [41].

Besides their beneficial effects on disease control (Table-1) [41–62], plants can also improve production yields [42]; stimulate appetite [63] and the immune system [43, 64]; improve integral health [65]; exert anti-inflammatory [44], antistress [66], sedative, and anesthetic effects [67]; and prolong the shelf life of aquatic products [68]. Moreover, there are numerous therapeutic plants worth mentioning because they are used to treat bacterial and parasitic diseases affecting vital organs, such as the skin, gills, gut, and eyes [41, 45, 69].

**Table-1:** Medicinal plants used in the production and processing of aquatic species and products.

Plant species	Function	Aquatic species	Form	Reference
Productive stage				
*Urtica dioica*	Improves productive yields Immunostimulant	*Oncorhynchus mykiss* *C. auratus*	Powder Methanolic extract	[43]
*Moringa Oleifera*	Antibacterial Immunostimulant Improves productive yields	*Carassius auratus gibelio*	Powder	[42]
*Eugenia aromatica*	Anesthetic	*Amphiprion ocellaris*	Essential oil	[46]
	Anesthetic and sedative	*C. carpio*		[47]
*Vitex agnus-castus*	Immunostimulant	*C. auratus*	Hydro-ethanolic extract	[48]
*Azadirachta indica*	Antiprotozoal	*Anabas testudineus*	Powder	[49]
	Antiparasitic	*C. auratus*	Ethanolic and aqueous extract	[50]
*Chenopodium ambrosioides*	Anti-inflammatory	*Lutjanus peru*	Powder Methanolic extract	[44]
*Curcuma longa*	Growth improvement Immunostimulant Antibacterial	*C. carpio*	Powder	[51]
*Origanum heracleoticum*	Antibacterial	*Ictalurus punctatus*	Essential oil	[52]
	Growth stimulant			
*Lippia berlandieri*	Antibacterial	*L. vannamei*	Essential oil	[53]
	Antibacterial	*Oreochromis mossambicus*	Essential oil	[54]
	Growth stimulant			
*Kalopanax pictus*	Antibacterial	*Epinephelus bruneus*	Ethanolic extract	[41]
*Scutellaria baicalensis*	Antibacterial	*Oplegnathus fasciatus*	Hydro-ethanolic extractw	[41]
*Mentha piperita*	Antibacterial	*Lates calcarifer*	Powder	[55]
	Growth stimulant			
	Antiparasitic	*Oreochromis niloticus*	Essential oil	[56]
*Caesalpinia sappan*	Antiparasitic	*C. auratus*	Methanolic extract	[45]
*Lysimachia christinae*				
*Cuscuta chinensis*				
*Artemisia argyi*				
*Eupatorium fortune*				
*Cinnamomum cassia*				
*Lindera aggregate*				
*Pseudolarix kaempferi*				
*Piper longum*				
*Bupleurum chinensis*				
*Andrographis paniculata*	Growth stimulant and Immunostimulant	*L. vannamei*	Powder	[57]
*Solanum procumbens* Lour	Immunostimulant			[58]
*Bidens alba*	Improves productive yields Immunostimulant Antibacterial			[59]
*Plectranthus amboinicus*				
*Eleutherine bulbosa*	Immunostimulant Antibacterial			[60]
Processing stage				
*Citrus sinensis*	Product preservation	*Parapenaeus longirostris*	Essential oil	[61]
*Salvia officinalis*	Increase in the shelf life of fish burgers	*C. carpio*		[62]

*C. auratus=Carassius auratus, C. carpio=Cyprinus carpio, L. vannamei=Litopenaeus vannamei*

## Garlic

*Allium* (Family: *Amaryllidaceae*) is a monocot genus having 800 species that are globally distributed, with different morphological and psychological characteristics [70]. Garlic has been used as a seasoning in food preparations and in traditional medicine to improve human health [70]. At the economic and commercial level, *A. sativum* is the most relevant species of the genus [71] and it is the most commonly used *Allium* species to treat common diseases, after onion (*Allium cepa* L.) [72].

Garlic has been shown to have antifungal, antibacterial, antiviral, antitoxic, and anticancer properties, among others [73–77]. Sulfur-containing bioactive compounds and sulfur-free phenolic compounds are responsible for these properties [63].

The proximate composition of garlic is 65% moisture, 27.5% carbohydrate, 4.7% fiber, 2%–3% organosulfur compounds, and 2% protein [78]. The sulfur-containing chemical compounds include ajoenes, thiosulfinates, vinilditine, sulfides, diallyl trisulfide, and cysteine [79]. Garlic also contains S-propyl-cysteine-sulfoxide and S-methyl cysteine-sulfoxide, which can produce more than 50 metabolites depending on the moisture content and temperature [80]. The secondary metabolites obtained from the cysteine accumulated in plants of the *Allium* genus are S-alqu(en)yl-cysteine sulfoxides; alliin, which transforms into allicin; N-acetylcysteine; S-allylcysteine; and S-allyl-mercapto cysteine [80, 81]. These active principles have antioxidant, anti-inflammatory, and anticancer properties [82].

Allicin is an unstable, volatile, and cytotoxic liposoluble organosulfur compound [83]. It is also garlic’s most important active component, with antiseptic, antiviral, antifungal, antiparasitic, and antibacterial properties [63, 84]. In addition, allicin and ajoene are the active components used in veterinary medicine and livestock production [85, 86], and their benefits and antiparasitic properties on the zootechnical performance of farm animals have been reported [63, 78–88].

The beneficial properties of garlic for the treatment of bacterial diseases caused by Gram-positive and Gram-negative bacteria are attributed to several thiosulfinates, including allicin [89, 90]. Garlic’s active components oxidize bacterial proteins [91] by reacting with low molecular weight thioles, such as glutathione, and changing the cellular redox potential into a more oxidized state, which induces cell apoptosis [92]. In addition, allicin’s lipophilic properties enhance its antimicrobial activity, allowing it to enter the cell through the bacterial cell wall [93].

Garlic also presents antiviral activity [94]. Allicin condenses into ajoene, a molecule with antiviral activity that blocks integrin-dependent processes in the infected cellular system [95]. Furthermore, allicin efficiently inhibits viral replication and stimulates the immune system through the host proteome [96], improving the host immune response through mechanisms involving interferon-gamma, tumor necrosis factor, interleukin (IL-12P70), and T-cells [83]. Moreover, another advantage of garlic is immunostimulation, which is attributed to organosulfur, polysaccharide, and fructan compounds [97].

## The Use of Garlic in Aquatic Organisms

Garlic has been used in livestock production primarily to increase the indicators of zootechnical performance [98]. In aquaculture, garlic has been used for the same purpose [99, 100]. Its effects on zootechnical performance have been studied in different species of aquatic organisms [29]. Various garlic species have been used in aquaculture, for example, the alcoholic extract of *Allium hirtifolium* was used in feed of rainbow trout *Oncorhynchus mykiss* fingerlings [101]; this species has also been studied in *Cyprinus carpio* [102]. Other species, such as *Allium stipitatum* [63], *Allium tuberosum* [103], and *Allium mongolicum* [104], have been studied in the production of aquatic organisms.

Reported results and effects are different, for example, when the food formulation incorporated garlic (in a proportion of 40 g/kg), Asian sea bass (*Lates calcarifer*) grew faster than fish that were fed commercial diets [105]. Grouper (*Epinephelus coioides*) that was fed garlic supplemented diet (80 g/kg) and exposed to *Vibrio alginolyticus*, presented a significant increase in phagocytosis, superoxide anion production, and superoxide dismutase activity, indicating that garlic can increase the resistance against bacterial diseases and stimulate the immune system [102]. In addition, these values were higher in the groups that received a fresh garlic diet than in those that were given garlic powder [106]. Diets containing garlic (10 g/kg of food) increased resistance to *Edwardsiella tarda* infection in African sharptooth catfish (*Clarias gariepinus*) [107]. In narrow-clawed crayfish (*Postantacus leptodactylus*), the use of garlic powder included in the feed (1%–2%) has effects on growth and immunostimulant effects [108].

One of the most reported effects of garlic consumption on fish is its antiparasitic properties, which are achieved primarily by subjecting the aquatic organisms to immersion baths [109]. The effects of garlic extract on adult and oncomiracidia, production, development, and cumulative hatching of eggs of *Neobenedenia* spp. did not yield positive results *in vitro* [110]. However, another *in vitro* study demonstrated that 250 μL/L of aqueous garlic extract prevented the mobilization of *Lernantropus kroyeri*, which was isolated from European bass (*Dicentrarchus labrax*), for 60 min [111].

Furthermore, *in vivo* studies performed on *Poecilia reticulata* infected with *Cryptocaryon irritans* showed that an immersion bath containing garlic aqueous extract (250 or 500 μL/L) could reduce infection, particularly in the caudal fin [112]. In addition to the immersion baths, adding aqueous garlic extract (50 mL and 150 mL/kg) to the diet of *L. calcarifer* for 30 days reduced *Neobenedenia* spp. infection by 70% [113].

## Garlic in Pisciculture

**Figure-1:**
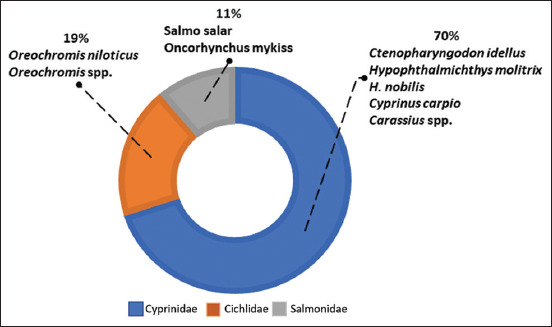
Primary fish species contributing to global fish farming production. Source: FAO, 2020.

Pisciculture is a subset of aquaculture, which encompasses the production of all aquatic organisms. It is a collection of techniques and knowledge related to industrial fish farming. The Food and Agriculture Organization [9] has registered the production of 466 aquatic species, 92 genera, and 32 families for fish farming. However, global fish farming production mainly relies on nine species, five of which are carps, two of which are tilapia, and the rest are salmonids (Figure-1).

### Carps

Carps belong to the *Cyprinidae* family, which includes important species for aquaculture due to their commercial value and distribution [114]. In the report of the Food and Agriculture Organization of the United Nations (FAO) 2020 [9], they composed 32% of the global freshwater aquaculture production.

Several factors can impact productive performance, such as the activity of digestive enzymes, structure, and gut microbiota [115]. Therefore, the implementation of products to improve nutrient absorption in fish has been thoroughly studied [116]. In this regard, it has been reported that adding ginger to the diet of the common carp (*C. carpio*) increases productive performance, which was attributed to an improvement in the digestion and absorption of dietary protein components due to the stimulation of gut protease secretion [117].

The use of garlic powder (0.5%, 1.0%, and 1.5% proportion) to supplement the diet of juvenile carps showed significant beneficial effects on weight gain, specific growth rate, and protein efficiency compared with the group that did not receive garlic powder-containing diet [118]. Moreover, adding 0.5% garlic powder to the diet of *Carassius auratus* improved the growth and survival of the juveniles [119].

In *Ctenopharyngodon idella*, the addition of 2% garlic powder in the diet significantly improved weight gain, food efficiency and food conversion indicators, and the percentage of protein in muscle when compared to fish with commercial diets without garlic powder [120]. Moreover, Adineh *et al*. [100] reported that the addition of 0.2% garlic and selenium nanoparticles to the diet of *C. idella* improved the activity of digestive enzymes, including amylase, protease, and lipase, which favored food assimilation and the subsequent improvement of growth indicators.

The immune system of fish is also affected by garlic. In *C. carpio*, the supplementation of commercial diets with garlic (5 g/kg) had a positive effect on the white cell count, especially on lymphocytes, and presented significantly higher productions of lysozyme and immunoglobulin M than the control groups fed commercial diets alone [121]. Another study on the same fish species has shown that 20 g/kg of garlic in the diet stimulates leukocyte production and phagocytosis [122].

Carps are prone to infectious diseases, and their first protective barrier is the skin mucus. It has been observed that when the *C. auratus* was fed a diet supplemented with 15 mL/kg of liquid garlic extract presented higher lysozyme, complement and alkaline phosphatase activity, and immunoglobulin and dissolved protein concentrations in skin mucus than the control groups. These results are reflected in their ability to inhibit the growth of *Aeromonas hydrophila*, *Yersinia ruckeri*, *Micrococcus luteus*, *Streptococcus faecium*, and *Streptococcus iniae* [123]. Using a commercial additive containing 2% garlic extract in the diet of *C. carpio* improved their resistance to experimental infection by *A. hydrophila*, resulting in a 100% survival rate compared to the 83% in the group that received the non-supplemented diet.

Ectoparasitic and endoparasitic infestations are common in carps [124]. *C. carpio* infested with *Gyrodactylus elegans* (Monogenean) and subjected to an immersion bath containing 8.37 mg/mL of garlic powder for 3 min reduced the parasite load by 14%. Moreover, 6 min of immersion with the same garlic concentration resulted in a 100% reduction of the parasite load [125].

### Tilapia

Garlic has been known to promote the growth of aquatic organisms [125]. The supplementation of balanced commercial food with 14 g/kg of garlic for juvenile tilapia resulted in an increase in weight and average length; supplementation with 10 g/kg of garlic resulted in decreased conversion factor [126]. *Tilapia zillii* juveniles fed with a basal diet supplemented with garlic powder (20 g/kg) showed a weight gain of 32 g/fish and a food conversion factor of 1.27. In contrast, the control group only gained 22 g/fish and presented a conversion factor of 1.55 [127].

Replacing basic dietary ingredients with garlic leads to results contrary to those previously highlighted. The substitution of corn flour with 1.5% of garlic powder in the diet of *Oreochromis niloticus* juveniles (initial weight of 10 g) did not significantly alter the growth indicators or the food conversion factor when compared with the control group [128]. Furthermore, it is essential to note that a previous study revealed that the addition of garlic does not modify the proximate composition of the final product, which remained identical to that of the control group [128].

Studies have shown that as garlic concentration in food increases, the growth-promoting properties of natural garlic extract become evident. With an initial weight of 7 g, *O. niloticus* gained approximately 15 g/fish after consuming a diet containing 3% garlic powder. In contrast, the group that did not consume garlic gained 11 g [129]. This study also reported that 4% garlic powder in the diet has no negative impact on the fish’s health and does not disrupt the activity of the alanine transaminase and aspartate aminotransferase enzymes; these results can be attributed to *A. sativum*, which may cause stabilized cell membrane and protect the liver against deleterious agents and free radical-mediated toxic damages to the liver cells. This is reflected in the reduction of liver enzymes. *A. sativum* helps the liver to maintain its normal function by accelerating the regenerative capacity of its cells [129].

The addition of 4% garlic in the diets of *O. niloticus* revealed positive health effects. Mesalhy Aly *et al*. [130] demonstrated that in juveniles, garlic provides resistance against *A. hydrophila* and *Pseudomonas flourescens*. Furthermore, adding 1% garlic to the diet of *O. niloticus* juveniles increases their gut microbiota activity, favors resistance against experimental *Streptococcus iniae* infection, and reduces symptoms of diseases, such as exophthalmia, erratic swimming, and changes in pigmentation of the head. It also decreased the cumulative mortality rate, which was below 20% in the treated group and 80% in the control group [131].

In *O. niloticus*, experimentally infected with *A. hydrophila*, immersion baths containing 1%, 1.5%, and 2% liquid garlic extracts yielded positive results, indicating that immersion is an efficient method for using garlic to counteract the effect of bacterial diseases in tilapia. Moreover, garlic can be used to treat bacterial diseases, as well as parasitic diseases.

Abd El-Galil and Aboelhadid [132] performed culture experiments and studies under laboratory conditions to determine the effects of different methods of garlic administration to treat *Trichodina* and *Gyrodactylus* infestations (Table-2). It has been observed that a concentration of 800 ppm of raw garlic was effective in eliminating *Trichodina* spp. in *O. niloticus* juveniles weighing 3.6 g. However, the infection recurred after 14 days, possibly due to the organic matter produced by the garlic residue, which allows the parasites to survive in the culture water [133].

**Table-2:** *In vivo* antiparasitic activity of garlic (*Allium sativum*).

Conditions	Method	Concentration	Immersion time	Results
Laboratory	Therapeutic: Garlic oil immersion	2, 2.5, 3 ppm	24 h	After 4 h of immersion, 100% of the parasites were eliminated.
		1, 1.5 ppm	24 h	After 4 h of immersion, 100% of the parasites were eliminated.
Culture	Therapeutic: Garlic oil immersion	3 ppm	1 h (bath before seeding)	74%, 76%, and 55% of juveniles were free of parasites after 1 h, 14 h, and 7 days, respectively. Group without garlic=24% parasite-free juveniles.
	Therapeutic: Macerated fresh garlic packed in permeable bags and added to the tank	300 g/m^3^	During the entire culture	31%, 79%, and 68% of juveniles were free of parasites after 1 h, 14 h, and 7 days, respectively.
Culture	Preemptive: Garlic oil immersion	3 ppm	1 h (bath before seeding)	Both treatments prevented parasite infection. Macerated garlic showed the best pre-emptive results.
	Preemptive: Macerated fresh garlic packed in permeable bags and added to the tank	300 g/m^3^	During the entire culture	

Source: Abd El-Galil and Aboelhadid [132]

### Salmonids

The addition of garlic powder (5 and 10 g/kg) to the balanced food of *O. mykiss* improved their weight gain percentage, which increased as the concentration of garlic increased. In addition, this study found that the group on a supplemented diet (10 g/kg) had a daily weight gain of 2 g/fish and a food conversion factor of 1.1, whereas, in the control group, these values were 1.2 and 2.3 g/fish, respectively [134].

The effects of garlic extract could be related to the activity of the gut microbiota because it has been reported to increase the operational taxonomic units of *Aeromonas*, *Deefgea*, *Exiguobacterium*, and *Mycoplasma*. These bacterial species have been shown to improve intestinal absorption, resulting in improved growth indicators of fish fed commercial balanced food supplemented with 2% liquid garlic extract [135].

The efforts made to incorporate garlic into the diets of *O. mykiss* have led to microencapsulation studies, a methodology that aims to improve the properties of bioactive components. Microencapsulation of 2% garlic in trout diets improves their growth performance and decreases the food conversion factor compared to the control group; the effects of garlic on animal growth may be related to *allicin*; and garlic has been reported to induce intestinal villi growth and digestive enzyme activity, which may improve fish growth [100].

The experimental group of *O. mykiss* species fed a 5 and 10 g/kg garlic-supplemented diet were more resistant to the bacteria *A. hydrophila* compared to the control group, showing survival rates of 86% and 16%, respectively [129]. Similarly, *O. mykiss* juveniles fed garlic-supplemented food (5 and 10 g/kg) and subjected to experimental infection with *Aeromonas salmonicida* were more resistant to the infection compared to the control group and showed a significant increase in circulating lymphocytes [136]. This is because the active ingredients of garlic stimulate the production of defense cells and increased lymphocyte concentration indicates that there is an increased inflammatory response, that is, cell-mediated and/or humoral immunity [136].

Garlic has been used in pisciculture to treat systemic bacterial diseases and to decrease the activity and growth of *Listeria* spp. in raw *Salmon salar* intended for human consumption without modifying the sensory properties of the product [137]. Furthermore, garlic oil decreases the bacterial load of *Salmonella enteritidis* and *Listeria monocytogenes* during the storage of *S. salar* at 2°C [138].

Studies performed in *S. salar* production farms showed that garlic supplementation (10 g/kg) reduced sea lice infestation (*Caligidae*: *Crustacea*) [139]. In *in vitro* assays, liquid garlic extract (16.9 mg/mL) eliminates 100% of amoebas, which infest the gills and skin of *O. mykiss* [140]. Garlic is also effective against the parasite *Ichthyophthirius multifiliis*, with a concentration of 62 mg/L in water eliminating the theront stage of the parasite in 15 h, and concentrations of 117 and 570 mg/L eliminating the tomont stage of the parasite in 24 h [141].

### Crustaceans

9.4 million tons of crustaceans are produced globally [9]. Similar to fish production, crustacean production systems are being expanded to contribute to food security and meet the demand for animal protein [12]. Therefore, crustaceans are a fundamental part of aquaculture. It is necessary to identify alternatives that will improve productivity indicators and can be used to prevent and treat bacterial and parasitic diseases while attempting to reduce the associated environmental impact [12].

Garlic has been used as a natural alternative to promote crustacean growth. Studies performed in *Macrobrachium rosenbergii* have shown that the addition of 1% garlic to their diet resulted in a higher survival rate (82%) and lower food conversion factor than the control group (66%), probably because the final weight of the treated and control groups was 2.33 g and 1.76 g, respectively [142]. Supplementation with garlic powder at 1% and 2% in the diet for narrow-clawed crayfish (*P. leptodactylus*) increased the growth indices and digestive enzymes activity, 2% elevated lysozyme, nitric oxide synthase, and phenoloxidase activities in the crayfish [108].

The addition of 2% garlic to the diet of *Procambarus clarkii* juveniles resulted in a weight gain of 1.2 g, whereas the control group gained 0.95 g. The study indicated that the best food conversion factor was found at concentrations of 2% and 3% garlic and also highlighted that the survival rate of the treated group was 50% higher than that of the control group [143]. Furthermore, Malar and Charles [144] observed that 2 months after adding 2% garlic concentration to the diet of *Penaeus monodon*, the survival rate increased from 77% (control group) to 87%. *P. monodon* showed similar growth improvement, and a 2% garlic concentration was reported as the optimal experimental concentration for the 1^st^ and 2^nd^ months of the study.

In whiteleg shrimp (*Litopenaeus vannamei*), dietary replacement with fish, meat, or bone flour with 3% garlic, improved growth and proximate composition even after 50% of the fish flour was replaced. These results indicate that garlic improves food consumption and the efficiency of utilization of proteins and amino acids [95]. The addition of 4% garlic for 2 months has been reported to have positive effects; however, a 6% concentration results in growth compared to the control group after 63 days of treatment [145]. Moreover, when compared to the control group, a 2% concentration has a positive effect on whiteleg shrimp growth, as do lower garlic concentrations (0.5% and 1%). This concentration also increased the conversion efficiency and, as a result, the food conversion factor; these effects might be due to an improvement in the immune system of the shrimp with a diet comprising 2% garlic concentration [146].

Among the diseases developed by shrimp, we found those caused by the white spot syndrome virus (WSSV) and *Vibrio parahaemolyticus*. It has been observed that shrimp fed with a 0.16% garlic-supplemented diet and exposed to WSSV and *V. parahaemolyticus* showed the highest survival rate after experimental infection [147]. Increasing the food garlic concentration to 2% and 4% for 30 days and exposing the crustaceans to *V. parahaemolyticus* improves their survival rate compared to the control group. This result could be attributed to the higher activity of superoxide dismutase and catalase observed in the treated group, as well as their ability to eliminate free radicals [148].

In *in vitro* experiments, liquid garlic extract at concentrations of 12.5%, 25%, and 50% reduced the growth of Gram-positive bacteria isolated from tiger shrimp (*P. monodon*), demonstrating the potential of garlic as a natural alternative in treating Gram-positive infections [149]. Furthermore, garlic exerts antiparasitic effects, for example, in *L*. *vannamei* infected by gregarines, the addition of mashed garlic (40 and 50 g/kg) to the diets decreased parasite load. Although it could not eliminate all the parasites, it controlled the infection [150, 151].

## Conclusion

Several studies have evaluated garlic’s properties as a food supplement and as an extract for immersion baths used in the production of aquatic organisms, both in pisciculture and crustacean production. In addition, there is particular interest in research on certain fish species such as *O. niloticus*, *C. carpio*, and *O. mykiss*, as well as crustaceans such as the whiteleg shrimp.

Most studies focus on demonstrating garlic’s properties as a growth promoter that can directly impact productive performance indicators by improving food consumption and efficiency as well as the food conversion factor. These studies also highlight the high survival rates observed in aquatic organisms that consumed a garlic-supplemented diet after a bacterial or parasitic experimental infection.

The concentrations of garlic used to supplement food and that of liquid garlic extracts for immersion baths vary depending on the application and desired effect. However, most studies recommend concentrations between 2% and 4% both for food supplementation and immersion baths to treat animals infected with parasites, and these studies agree that high concentrations could cause harmful effects or may not benefit aquatic organisms.

Due to its properties and active components, garlic has become an effective alternative for use in the production of aquatic organisms in both continental water and seawater to improve productive performance indicators, increase bacterial resistance, and treat parasitic diseases. In addition, garlic has other functions and properties that this review could not describe in detail, such as immunostimulation or immunomodulation, antioxidant properties, stress-reduction abilities, and antiviral activity.

## Authors’ Contributions

DLCD, SACG, and ADO: Conceptualization. DLCD and LLCC: Data curation. DLCD, LLCC, and ADO: Formal Analysis. DLCD: Funding acquisition. DLCD, SACG, and ADO: methodological implementation. DLCD, SACG, and LLCC: Drafted and revised the manuscript. All authors have read, reviewed, and approved the final manuscript.
